# EHMTI-0277. Monitoring the use of symptomatic drugs in headache patients: a 6 month follow-up

**DOI:** 10.1186/1129-2377-15-S1-D74

**Published:** 2014-09-18

**Authors:** C Voiticovschi-Iosob, F Antonaci, L Gervasio, C Fattore, M Bianchi, I De Cillis, G Nappi, N Vanacore

**Affiliations:** 1 Headache Center, C. Mondino National Institute of Neurology Foundation IRCCS Pavia Italy and State Medical and Pharmaceutical University “NicolaeTestemitanu”, Chisinau, Moldova; 2 Headache Center, C. Mondino National Institute of Neurology Foundation IRCCS and Dept. of Brain and Behavioral Sciences University of Pavia, Pavia, Italy; 3 Pharmaceutic Service, C. Mondino National Institute of Neurology Foundation IRCCS, Pavia, Italy; 4 Clinical Trial Center and Antiepileptic Drugs, C. Mondino National Institute of Neurology Foundation IRCCS, Pavia, Italy; 5 Headache Center, C. Mondino National Institute of Neurology Foundation, Pavia, Italy; 6 Headache Center, C. Mondino National Institute of Neurology Foundation IRCCS, Pavia, Italy; 7 Headache Center, C. Mondino National Institute of Neurology Foundation IRCCS and Dept. of Brain and Behavioral Sciences, Pavia, Italy; 8 Statistics, Istituto Superiore di Sanità, Roma, Italy

## Background

Headache is an extremely common neurological problem. Italy is the first European country for OTC consumption with related problems of self-medication and risk of medication overuse headache (MOH).

## Aim

to monitor the consumption of symptomatic drugs for headache and to prevent drugs abuse/dependence.

## Materials and methods

274 patients using symptomatic drug for headache were recruited in 32 pharmacies in the Pavia Health District. A telephonic interview was carried out in 199 patients; 179 entered the study at baseline (T0) and 112 (22 M and 90 F, mean age 45.0±11.5 yrs.) were followed-up at 6 months (T6).

## Results

patients with chronic migraine or MOH at T0 were 39 and 7 at T6. Days/month with headache at T6 vs T0 were 4.3±0.6 vs 9.7±0.8 (p<0.0001). Attacks/month at T6 vs T0 were slightly reduced (1.9±0.2 vs 7.6±0.8 p=0.09). A significant decrease of the doses of analgesics consumption/month was noted (T6=13.2±1.2 vsT0=17.0±2.2, p=0.013). An increase in quality of life was found on MIDAS scores at T6 vsT0 (13.4±1.8 vs23.7±2.5; p=0.00) and in the quality of treatment received (HURT)(5.6±0.4 vs 9.9±0.5; p=0.00).

## Conclusions

Our results highlight that the change from self medication to medical care may reduce the numbers of symptomatic treatment, the headache days/ month and ameliorate the quality of life in patients with headache. A longer follow-up (i.e. 12 month) may provide further evidence on improvement of the clinical picture of headache patients and prevention of MOH.

No conflict of interest.

